# Primary Hydatid Cyst of the adrenal gland: A case report and a review of the literature

**DOI:** 10.1016/j.ijscr.2020.04.073

**Published:** 2020-05-07

**Authors:** Skander Zouari, Chakroun Marouene, Hana Bibani, Ahmed Saadi, Anis Sellami, Linda Haj Kacem, Ahlem Blel, Abderrazek Bouzouita, Amine Derouiche, Riadh Ben Slama, Soumaya Rammeh, Haroun Ayed, Mohamed Chebil

**Affiliations:** a Urology Department, Charles Nicolle Hospital, Tunis, Tunisia; b Radiology Department, Charles Nicolle Hospital, Tunis, Tunisia; c Pathology Department, Charles Nicolle Hospital, Tunis, Tunisia

**Keywords:** Adrenal gland, Hydatid cyst, Unusual location, Surgical approach

## Abstract

**Introduction:**

In North Africa which is an endemic region for Hydatid Cyst, Tunisia is considered as an endemic country. The liver and lungs are common locations for Hydatid Cysts, whereas the Adrenal Glands are unusual and rare locations.

**Presentation of case:**

Here is a report of primary Hydatid Cyst in a 55-year old patient, with left hypochondrium pain as chief complain. No remarkable findings were revealed by physical examination and blood analysis showed normal range. Hydatid serology was negative. The diagnosis of Hydatid Cyst was suspected based on CT Scan results which showed a well-circumscribed, non-enhanced, multi-cystic, 12 cm mass with scattered calcifications located in the left adrenal gland. Therefore, the patient underwent an open surgery with resection of the protruding dome of the cyst as it was attached to the renal pedicle, the pancreatic tail, the spleen and the jejunum. The final pathological examination of the specimen led to a Hydatid Cyst.

**Discussion:**

Throughout an extensive literature review that we have made, we have analyzed 54 reported cases, with their clinical presentations, biological exams, radiological features and surgical managements. The treatment should be surgical and has to be as conservative as possible. The prevention of the parasite transmission has to be the cornerstone of the disease management.

**Conclusion:**

The hydatid cyst of the adrenal gland remains a rare diagnosis that has to be evoked in case of an adrenal gland cyst, especially in an endemic country.

## Highlights

• Adrenal gland is an unusual location for hydatid cyst.
• Clinical presentation and physical examination are unspecific, and can not raise the diagnosis.
• Imaging is based on ultrasound, and CT scan, can suspect the diagnosis, describing precisely the features of the hydatid cyst.
• Only pathological examination of the specimen after surgery can confirm the diagnosis.
• The treatment is surgical and has to be as conservative as possible.

## 1 Introduction

The hydatid cyst is a well-known zoononis that is endemic in some areas of the world, such as North Africa and Middle East, Central Asia, Australia and Latin America [1]. It is caused by Echinococcus granulosus larva, after being infested with the parasite through ingestion of contaminated water or vegetables, or direct contact with dogs. Common locations of the hydatid cyst are the liver and the lungs. Adrenal gland is an unusual and extreme rare location of the hydatid cyst, especially when it is the primary location. Herein we report a case of a primary hydatid cyst of the adrenal gland. Clinical radiological features will be discussed as well as the surgical procedure with a literature review of the previous cases. The work has been reported in line with the SCARE criteria [2].

## 2 Case report

**Fig. 1:**
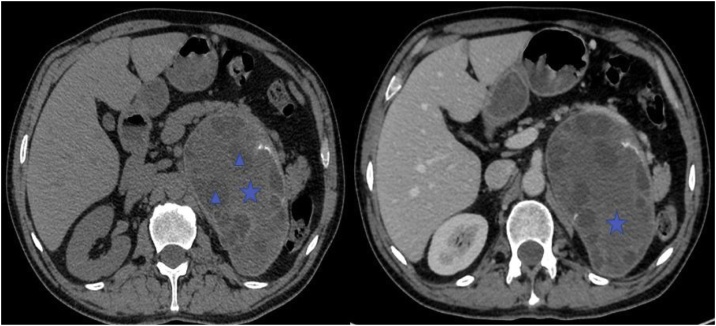
Axial unenhanced and contrast-enhanced axial CT showed a cystic echinococcus 3b of the WHO classification: unilocular cystic well defined cystic mass with daughter cysts  inside a mucinous or solid cyst matrix  (high density region). No significant enhancement has been detected in any part of the cyst.

**Fig. 2:**
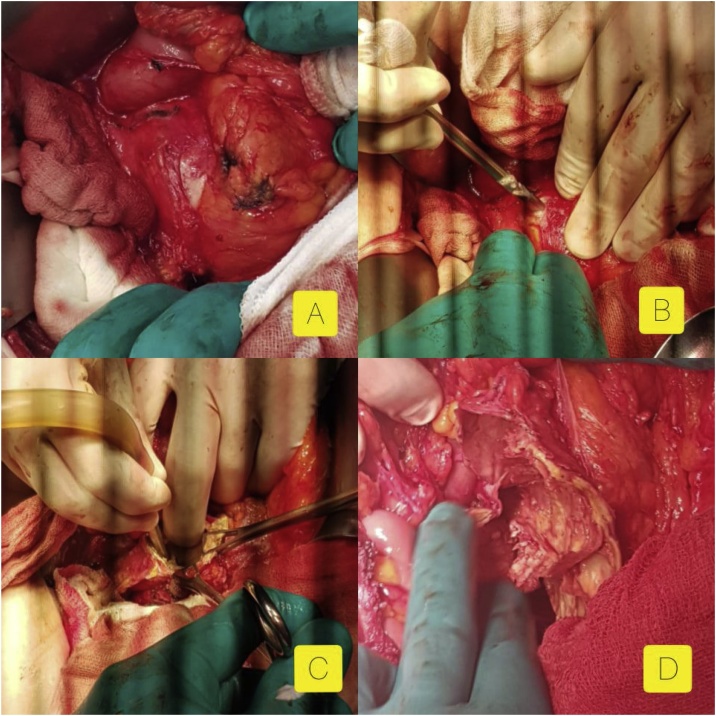
A. The adrenal cyst, contiguous to the tail of the pancreas. B. Resection of the protruding dome, after sterilizing the content of the cyst. C. Aspiration of the content of the cyst. D. The aspect of the residual cavity during the resection of the protruding dome.

**Fig. 3:**
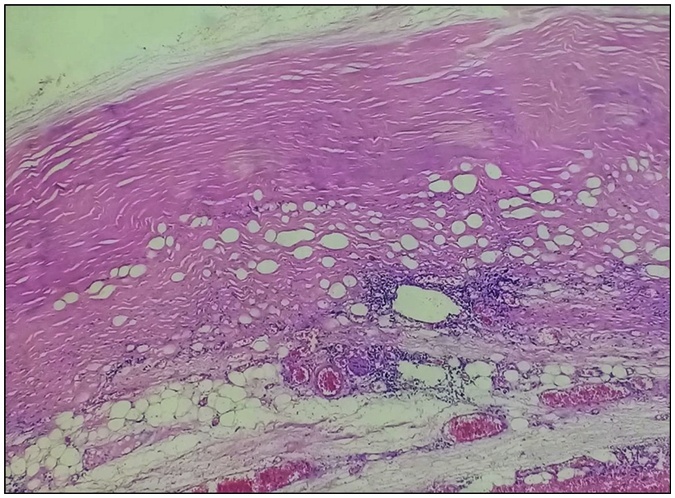
Hydatid periscyst: histological aspect of a thick fibrous and fatty cystic wall containing monomorphic inflammatory elements (×10 magnification).

A 55-year-old patient, living in an endemic area, was referred to our institution for pain in the left hypochondrium evolving for 6 months, with no other symptoms such as: nausea and vomiting. Arterial blood pressure was within normal limits. No past medical history was reported. No anomalies were detected in the physical examination. Blood analysis including complete blood count, 24-h urinary VMA and metanephrine were normal. Serological analysis of the blood was negative for Echinococcus IgM and IgG. An abdominal sonography showed a large hypoechoic retroperitoneal mass in the upper pole of the left kidney containing internal cystic component. The CT scan showed a well-circumscribed non-enhanced multi-cystic 12 cm mass with scattered calcifications located above the left kidney and the left adrenal gland which seemed shadowed (Fig. 1). The mass has a compressive effect on the adjacent organs with no perirenal or adrenal fat infiltration, no invasion of thick wall blood vessels and no extension beyond renal lodge, suggesting the diagnosis of hydatic cyst. There were no other locations, particularly in the liver or the lungs. Subsequently, the patient underwent a surgical removal of the mass by an open laparotomy by a left subcostal incision. Intraoperatively, we found a cyst measuring 12 cm in its largest diameter, contiguous to the renal pedicle, the pancreatic tail, the spleen, and the jejunum. Its wall colour was pearly white. We could surgically release the mass from the kidney and the jejunum, but the dissection along the pancreas was dangerous. Thus, we performed a resection of the protruding dome, after sterilizing the content of the cyst (Fig. 2). The puncture of the cyst showed a multivesicular clear content. The postoperative course was uneventful and the patient was discharged on the 3rd day postoperatively. The final pathological examination of the specimen led to a hydatid cyst of the adrenal gland (Fig. 3).

## 3 Discussion

Hydatid cysts are usually found in the liver or the lungs. It occurs when humans ingest accidentally echinococcus eggs found in the dogs’ stools. After passing through the digestive system, the larvae are released; they pass through hepatic filter to the lungs. If they are not destroyed by the immune system or trapped in the filtration system; they can spread anywhere in the body, giving unusual locations of the hydatid cyst as spleen, kidneys, brain, bones, heart, muscle tissue, pancreas, retroperitoneum, thyroid and adrenal glands, through the arterial circulation. Other mechanisms of the dissemination of the larvae imply entering the systemic circulation through lymphatic vessels, or by adjacent contact.

The adrenal location of the hydatid cyst is rare and accounts for less than 0.5% off all hydatid cysts [3,4]. It generally occurs in the context of a disseminated hydatid disease. In the other hand, hydatid cyst constitutes 7% of adrenal gland cysts. Isolated, primary adrenal hydatid cyst are extremely rare [5,6]. Most of the published cases are case reports. The largest series published in the literature was made by Ackay [7] in 2004 with 9 reported cases. In 2006, Horchani et al. have reported 6 cases over a 10 year period [8].

Based on a systematic Pubmed search using the keywords ‘Adrenal Gland’ and ‘Hydatid Cyst’, we have found 76 cases published in the literature. We have excluded those who could not be opened. We have selected therefore 54 cases for review. All the cases are summarized in (Table 1). The data has been analyzed using SPSS version 19.0 software. The main limitation of our analysis is the lack of information in some cases.

**Table 1:** Reported cases of hydatid cyst of the adrenal gland.

Author	Age	Sex	Environment	Contact with animals	Symptoms	Hypereosinophilia	Serology	Side	Size on imaging (cms)	Primary/Secondary	Treatment by albendazole	Surgical access	Follow up (months)	Recurrence
Fitzgerald EJ (1985)	48	M	N/A	N/A	Right hypochondruim pain	N/A	Positive	Right	18	Primary	N/A	Open surgery	N/A	N/A
Sroujieh AS (1990)	50	N/A	N/A	N/A	N/A	N/A	N/A	N/A	N/A	Secondary	N/A	Open surgery	N/A	N/A
Schoretsanitis G (1998)	48	M	Rural	Yes	Right hypochondruim pain	No	Negative	Right	9.5	Primary	No	Open surgery	N/A	N/A
Defechereux T (2000)	37	F	N/A	N/A	Left Flank Pain	No	N/A	Left	5	Secondary	No	Laparoscopy	N/A	N/A
C.Ö. Yeniyol (2000)	51	F	N/A	N/A	Left Flank pain	N/A	Positive	Left	6	Primary	N/A	N/A	N/A	N/A
el Idrissi Dafali A (2002)	28	N/A	Urban	No	Right hypochondruim pain	Yes	Positive	Right	5,7	Primary	No	Open Surgery	48	No
Escudero MD (2002)	40	F	Urban	No	Hypertension	N/A	N/A	Left	10	Primary	N/A	Open Surgery	12	No
Mufide Nuran Akcay (2003)	53	F	N/A	N/A	Right hypochondruim pain	N/A	Positive	Right	8	Primary	N/A	N/A	16	No
	80	M	N/A	N/A	Right hypochondruim pain	N/A	Positive	Right	10	Secondary	N/A	N/A	16	No
	48	F	N/A	N/A	Incidental	N/A	Negative	Right	4,5	Secondary	N/A	N/A	16	No
	61	F	N/A	N/A	Left hypochondruim pain	N/A	Positive	Left	6,5	Primary	N/A	N/A	16	No
	18	F	N/A	N/A	Incidental	N/A	Negative	Right	10	Secondary	N/A	N/A	16	No
	15	M	N/A	N/A	Right Flank pain	N/A	Positive	Right	12	Primary	N/A	N/A	16	No
	18	M	N/A	N/A	Left Flank pain	N/A	Positive	Left	20	Primary	N/A	N/A	16	No
	41	M	N/A	N/A	Right hypochondruim pain	N/A	Positive	Right	5	Secondary	Yes	N/A	16	No
	28	F	N/A	N/A	Incidental	N/A	Negative	Right	20	Primary	N/A	N/A	16	No
Gürdal M (2004)	48	F	N/A	N/A	Right Flank pain	No	Negative	Right	6	Primary	N/A	Open Surgery	12	No
Recai Gurbuz (2005)	47	F	N/A	N/A	Left Flank pain	N/A	N/A	Left	7,8	Primary	N/A	Open Surgery	Yes	N/A
H. Bedioui (2005)	20	M	N/A	N/A	Epigastralgia	No	Positive	Right	5,6	Primary	N/A	Open Surgery	24	No
	50	M	N/A	N/A	Left hypochondruim pain	No	Negative	Left	6	Primary	N/A	Open Surgery	36	No
Ali Horchani (2006)	24	M	N/A	N/A	Right Flank pain	N/A	Positive	Right	5,6	Primary	N/A	Open Surgery	24	No
	47	M	N/A	N/A	Left Flank pain	N/A	Negative	Left	8	Primary	N/A	Laparoscopy	24	No
	55	M	N/A	N/A	Right Flank pain + Hypertension	N/A	Negative	Right	6	Primary	N/A	Open Surgery	24	No
	59	M	N/A	N/A	Left Flank pain	N/A	Positive	Left	7	Secondary	N/A	Open Surgery	24	No
	54	M	N/A	N/A	Left Flank pain	N/A	Negative	Left	6	Primary	N/A	Open Surgery	24	No
	44	F	N/A	N/A	Right hypochondruim pain	N/A	Negative	Right	5	Primary	N/A	Open Surgery	24	No
Nikica Grubor (2006)	52	M	N/A	N/A	Epigastralgia	No	N/A	Right	4,4	Primary	N/A	Open Surgery	N/A	N/A
Ozarmagan S (2006)	54	F	N/A	N/A	Hypertension	N/A	Positive	Right	5	Primary	Yes	Open Surgery	N/A	N/A
Safioleas (2006)	61	F	N/A	N/A	Epigastralgia/Hypertension	Yes	Positive	Left	5,8	Primary	Yes	Open Surgery	6	No
Tsaroucha AK (2007)	56	M	Rural	Yes	Hypertension	No	Negative	Left	7	Primary	No	Open Surgery	12	No
Shintaro Maru (2007)	79	F	Urban	N/A	Impaired General Condition	No	Positive	Right	5,5	Primary	No	Open Surgery	N/A	N/A
Gianlorenzo Dionigi (2007)	68	F	N/A	Yes	Left Flank pain	No	Positive	Left	3	Primary	Yes	Laparoscopy	6	N/A
Ruiz-Rabelo JF (2008)	70	F	N/A	N/A	Left hypochondruim pain + Fever	No	Negative	Left	9	Secondary	No	Open Surgery	N/A	No
Tamotsu Kamishima (2009)	77	M	N/A	N/A	Hypertension	No	N/A	Right	6	Primary	No	Laparoscopy	N/A	N/A
O.Baraket (2010)	38	F	Rural	Yes	Left Flank pain	N/A	N/A	Left	7	Primary	N/A	Open Surgery	36	No
B Geramizadeh (2011)	49	F	N/A	N/A	Left Flank pain + Hypertension	No	N/A	Left	8,2	Primary	No	N/A	2	No
Limaiem F (2012)	55	F	N/A	N/A	Left hypochondruim pain	No	N/A	N/A	12	Secondary	N/A	N/A	N/A	N/A
Fadl Tazi (2012)	64	M	N/A	N/A	Left Flank pain + Hypertension	Yes	Negative	Left	14,5	Primary	Yes	Open Surgery	24	No
Maral Mokhtari (2012)	66	F	N/A	N/A	Right Flank pain + Hypertension	No	N/A	Right	5	Primary	No	Open Surgery	N/A	N/A
Huang M (2013)	45	M	N/A	N/A	Incidental	N/A	N/A	Right	9,5	Primary	No	Open Surgery	24	N/A
	56	F	N/A	N/A	Right hypochondruim pain	N/A	N/A	Right	11,2	Primary	No	Open Surgery	24	N/A
Abdulla Darwish (2013)	30	F	N/A	N/A	Hyperemesis gravidarum	No	Negative	Right	12	Primary	No	Openj Surgery	N/A	N/A
Babinska A (2014)	47	F	N/A	N/A	Hypertension	Yes	N/A	Right	6,8	Primary	N/A	Laparoscopic	132	No
Santosh Kumar (2014)	51	F	N/A	Yes	Left hypochondruim pain	No	Positive	Left	N/A	Primary	Yes	Laparoscopic	6	No
Afshin Mohammadi (2014)	N/A	M	N/A	N/A	Hypertension	N/A	N/A	Left	13	Primary	Yes	Open Surgery	6	No
Walter Nardi (2015)	55	M	N/A	N/A	Back pain	No	Negative	Left	6,5	Primary	N/A	Laparoscopic	N/A	N/A
Ammar Mahmoudi (2015)	76	F	Rural	N/A	Right hypochondruim pain	N/A	N/A	Right	5	Secondary	No	Open Surgery	24	No
Silke Spahn (2016)	78	M	N/A	N/A	Incidental	No	Positive	Right	7,4	Primary	Yes	Open Surgery	24	No
Gaurav Prakash (2016)	35	M	Rural	Yes	Right Flank pain	No	N/A	Right	16	Primary	Yes	Open Surgery	N/A	N/A
Fatehi Elnour Elzein (2016)	44	M	N/A	Yes	Right Flank pain	N/A	Negative	Right	10	Primary	Yes	Open Surgery	N/A	N/A
Sami Akbulut (2016)	64	M	N/A	N/A	Vague abdominal pain	No	Positive	Right	15	Primary	Yes	Open Surgery	24	N/A
Giovanni Aprea (2016)	78	F	Urban	No	Right Flank pain	No	N/A	Right	3,4	Primary	Yes	Laparoscopic	N/A	N/A
Ann-Katrin Seidel (2017)	16	M	N/A	N/A	Incidental	No	Positive	Right	7	Primary	Yes	Open Surgery	N/A	N/A
Sami Akbulut (2018)	64	M	Urban	No	Right hypochondruim pain	N/A	Positive	Right	7	Primary	Yes	Open Surgery	12	No
**Present case (2020)**	55	M	Urban	No	Left hypochondruim pain	No	Negative	Left	12	Primary	No	Open Surgery	12	No

N/A : not available.

Adrenal hydatid is more likely to be discovered during the 5th decade. The mean age in our review was 50 years. It occurred equally in men and women. It is most frequently discovered incidentally during surgery or on radiology, as autopsies reports shows an incidence between 0.06 and 0.18% with 92% of the lesions being unilateral [9]. Common presenting symptoms may include vague hypochondruim or flank pain, nausea and vomiting, or palpable mass. Rarely, it is discovered by arterial hypertension, resulting from compression of renal parenchyma in large cysts [10]. In our review, abdominal or hyponchondruim pain was the most presenting symptom in 66% of the cases. Arterial hypertension occurred in 17% while it was discovered incidentally in 11% of the cases. The right side was most affected in 62% of the cases. Biological exams could help raise the diagnosis, but they are unspecific. Hypereosinophilia was reported 4 times while hydatid serology was positive in 20 cases. Of those cases, the hydatid cyst was secondary to a previous location in 3 patients. In our patients, we did not find hypereosinophilia, and the hydatid serology was negative.

The radiological findings can orient the diagnosis. Ultrasound remains the first exam performed for this localization. The depth of the adrenal glands and sometimes peripheral calcifications make this exploration difficult. The computed tomography scan then allows for a better understanding of the location and relationships with the surrounding organs [11]. But often, the diagnosis assessment is made intraoperatively, as several other diagnoses can be evoked. Differential diagnosis may include cystic lymphangioma, pseudo haemorragic cyst, cyst with epithelial coating or extraadrenal cystic masses. The standard treatment of the hydatid cyst remains surgical.

The most recommended surgical procedure is the resection of the protruding dome with drainage of residual cavity. A pericystectomy of the hydatid cyst, or, if this is not possible, total excision of the adrenal gland can also be performed. Either laparoscopic or open procedure can be used [12,13], depending on the tumor size. Laparoscopic procedure is usually avoided, especially when the tumor size is above 6 cm and when radiological findings show daughter cysts within the cavity because of the risk of peritoneum spread due to insufflation [13]. In our review, laparoscopic method was used in 9 cases, and open procedure in 33 cases.

Laparotomy can be performed either by intercostal lumbar access with or without resection of the rib; or transperitonal anterior access allowing a sufficient view on the liver in case of associated localization. In our case, we performed a left subcostal incision, finding an accolated cyst to the pancreas, jejunum and renal pedicle. It is important to protect the operative field with compresses soaked in hypertonic (20%) sodium chloride solution to prevent larvae dissemination in case of accidental opening of the cyst intraoperatively. Conservative management of the adrenal gland is the standard attitude, except in the case of adrenal gland destruction by the cyst. In most of the cases, post-operative course is uneventful. Surgical removal of the hydatid cyst may normalise the hypertension if it was associated to the hydatid cyst preoperatively. We found normalization of the hypertension in 63% in our review. Among the published cases, no recurrence has been reported, as the case of our patient 10 months after the surgery. Post-operative albendazole prophylaxis is still controversial, even if some authors recommend it. The prevention of hydatic transmission remains an indispensable measure to avoid hydatid disease whatever his location is.

## 4 Conclusion

The hydatid cyst of the adrenal gland remains a rare diagnosis that has to be evoked in case of an adrenal gland cyst, especially in an endemic country. Clinical presentation and physical examination remain unspecific. CT Scan in combination with hydatid serology help raise the diagnosis, that is confirmed by the final pathological examination of the specimen. The treatment is surgical and has to be as conservative as possible. The prevention of the parasite transmission has to be the cornerstone of the disease management.

## Declaration of Competing Interest

The authors have no conflict of interest to declare.

## Sources of funding

This research did not receive any specific grant from funding agencies in the public, commercial, or not-for-profit sectors.

## Ethical approval

Given the nature of the article, a case report, no ethical approval was required.

## Consent

Written informed consent was obtained from the patient for publication of this case and accompanying images. A copy of the written consent is available for review by the Editor-in-Chief of this journal on request.

## Author contribution

- Skander Zouari: Writing - original draft
- Marouene Chakroun: Writing - review & editing.
- Hana Bibani: Writing - original draft.
- Ahmed Saadi: Writing - review & editing.
- Anis Sellami: Data interpretation of the radiological findings.
- Linda Haj Kacem: Data interpretation of the pathological findings.
- Ahlem Blel: Data interpretation of the pathological findings.
- Bouzouita Abderrazek: Project administration.
- Amine Derouiche: Supervision.
- Riadh Ben Slama: Study concept and design, data collection
- Soumaya Rammeh: Data interpretation of the pathological findings
- Haroun Ayed: Supervision; Reviewing and editing
- Mohamed Chebil: Supervision; Reviewing and editing

## Registration of research studies

This does not apply as it is a case report of a patient who has given written consent and has been de-identified. It is therefore not prospective research involving human participant.

## Guarantor

Dr. Skander Zouari.

## Provenance and peer review

Not commissioned, externally peer-reviewed.
